# Supplementary material for: STAN: spatio-temporal attention network for pandemic prediction using real-world evidence
Source: J Am Med Inform Assoc. 2021 Jan 22;28(4):733–43. doi: 10.1093/jamia/ocaa322 (PMC7928935; doi:10.1093/jamia/ocaa322)
Supplement: ocaa322_Supplementary_Data [file ocaa322_supplementary_data.zip › determination.pdf]

I ILLINOIS

OFFICE OF THE VICE CHANCELLOR
FOR RESEARCH & INNOVATION

Office for the Protection of Research Subjects
805 W. Pennsylvania Ave., MC-095
Urbana, IL 61801-4822

Notice of Not Human Subjects Research Determination

October 29, 2020

**Principal Investigator**	Jimeng Sun
**Protocol Title**	*Spatio-Temporal Attention Network for Pandemic Prediction Using Real World Evidence*
**Protocol Number**	21352
**Funding Source**	Unfunded
**Study Description**	A commercially-available dataset consisting of aggregated statistics of COVID-19 case counts at location (county level) at specific time (daily for 6 months), clinical variables (about 50 ICD-10 codes related to flu and COVID-19), and case counts will be purchased from IQVIA. The UIUC investigators do not have access to any identifiable private information, thus this project does not require IRB approval.
**Study Components**	Y N<br>☒ ☐ The project is a systematic investigation designed to contribute to generalizable knowledge.<br>☒ ☐ The project involves obtaining information about living individuals.<br>☐ ☒ The project involves interaction or intervention with human subjects or their identifiable private information.<br>☐ ☒ There is a way to access a code to re-identify coded data.<br>☐ ☒ The project involves human subjects as recipients of tests articles and/or medical devices.
**Determination**	It has been determined that this project, as described, does not meet the definition of Human Subjects Research as defined in 45CFR46(d)(f) or 21CFR56.102(c)(e) and does not require IRB approval.

The Office for the Protection of Research Subjects has reviewed and determined that the research study, *as described and reported to OPRS*, does not meet the criteria for Human Subjects Research. IRB approval is not required. This determination only applies to the research study as submitted. Please note that modifications may need to be submitted to OPRS for review, status determination, or approval before the modifications are implemented.

NHSR Determination

For Requesting a Written Determination that Activities are Not Human Subjects Research
All forms must be typewritten and submitted via email to irb@illinois.edu.

**When to Use this Form:** A researcher can complete and submit this form if they believe the activities they are engaging are not Human Subjects Research, as defined by federal regulations. Submitting this form to OPRS via email to irb@illinois.edu is a request for a written determination that the activities are Not Human Subjects Research (NHSR). Additional information may be requested as needed. If it is determined that the study is Human Subjects Research, an application will need to be submitted to OPRS for review.
OPRS uses decision trees to determine whether an activity is Human Subjects Research. These trees can be found here and may be helpful in thinking about your research.
If anything changes after a determination is made, please contact OPRS for another determination.

Section 1. PRINCIPAL INVESTIGATOR

Last Name: Sun	First Name: Jimeng	Degree(s): PhD	
Dept. or Unit: CS	Office Address: 201 N. Goodwin Avenue		
Street Address: 201 N. Goodwin Avenue	City: Urbana	State: IL	Zip Code: 61801
Phone: 2173336741	E-mail: jimeng@illinois.edu		
Urbana-Champaign Campus Status:<br>Non-visiting member of (Mark One) ☐ Faculty ☒ Academic Professional/Staff<br>(*Student Investigators cannot serve as PI*)			

Section 2. CO-INVESTIGATORS

**List additional co-investigators that should be CCed on correspondence from OPRS.**
Name:<br>Member of Urbana-Champaign Campus as: ☐ Faculty ☐ Academic Professional/Staff ☐ Student<br>Email Address:
Name:<br>Member of Urbana-Champaign Campus as: ☐ Faculty ☐ Academic Professional/Staff ☐ Student<br>Email Address:
Name:<br>Member of Urbana-Champaign Campus as: ☐ Faculty ☐ Academic Professional/Staff ☐ Student<br>Email Address:
Insert additional rows as necessary.

Section 3. PROTOCOL TITLE

Spatio-Temporal Attention Network for Pandemic Prediction Using Real World Evidence

NHSR Determination

Section 4. FUNDING

**4A. Is this research funded or pending funding?** ☐ Yes ☒ No	
**4B. If yes, who is the (potential) funder?**	

Section 5. STUDY ACTIVITIES

**Provide a response to each of the following questions. Indicate "N/A" if a question is not applicable.**
**5A. Describe the purpose, specific aims, and/or objectives of this project:**<br>developing a hybrid model for earlier and more accurate pandemic outbreak predictions
**5B. Does this project involve collecting primary data?** ☐ Yes ☒ No<br>***(Primary data is original data that will be collected specifically for this project.)***<br>**If "yes," complete Sections 5C-5E, then move to Section 6.**<br>**If "no," move to Section 5F.**
**5C. Describe what data will be collected, how, and by whom:**
**5D. Are you using any surveys, questionnaires, or interview guides?** ☐ Yes ☒ No<br>**If "yes," attach all such research materials with this submission.** ☐ Attached
**5E. Will the collected information be able to be directly or indirectly associated/linked with individual identities?** ☐ Yes ☐ No
**5F. Does this project involve using secondary data?** ☒ Yes ☐ No<br>***(Secondary data is data collected for a use other than the current project.)***<br>**If "yes," complete Sections 5G-5J, then move to Section 6. If "no," move to Section 6.**
**5G. Describe the secondary data/samples researchers will have access to, including names of datasets, URLs, etc.:**<br>Our collaborator from IQVIA will provide a dataset about aggregated statistics of case counts at location (county level) at specific time (daily for 6 months) and clinical variables (about 50 ICD-10 codes related to flu and COVID), and case counts. These statistics are computed from a claims dataset from IQVIA. We don't have access to the claims data but only the statistics.
**5H. How were data/samples originally gathered?**<br>193 counties
**5I. Does an identity key exist for this data?** ☐ Yes ☒ No<br>**If yes, will researchers be granted access to the identity key?** ☐ Yes ☐ No
**5J. Are the researchers and the organization providing the data/specimens entering into an agreement?** ☐ Yes ☒ No<br>**If "yes," attach all such agreements with this submission.** ☐ Attached

Section 6. DISSEMINATION

**6A. Describe how the information produced by this project will be disseminated:**<br>journal publication

NHSR Determination

**6B. Will individual identifiers be published, presented, or disseminated in other ways?**<br>☐ Yes ☒ No<br>**If yes, explain:**

Section 7. FORM COMPLETION

**Form completed by:** Jimeng Sun
**Date completed:** Oct 23, 2020
