# Supplementary material for: STAN: spatio-temporal attention network for pandemic prediction using real-world evidence
Source: J Am Med Inform Assoc. 2021 Jan 22;28(4):733–43. doi: 10.1093/jamia/ocaa322 (PMC7928935; doi:10.1093/jamia/ocaa322)
Supplement: ocaa322_Supplementary_Data [file ocaa322_supplementary_data.zip › Supplementary.docx]

# SUPPLEMENTARY

## DATA DETAILS

The data from JHU Coronavirus Resource Center was collected from March 22, 2020 to June 10, 2020. It has the number of active cases, confirmed cases, and deaths related to COVID-19 for different US locations. We select states with more than 1,000 confirmed cases by May 17 to ensure the data source accuracy, and finally we have 45 states and 193 counties in the dataset. For those counties, we set the number of cases before their respective first record dates as zero.

The IQVIA’s claims data is from the IQVIA US9 Database. The claims data are directly exported from the IQVIA's US claims database. This database contains adjudicated medical and pharmacy claims, including patient enrollment data for national and sub-national health plans and self-insured employer groups. We aggregated the hospitalization and ICU-related ICD codes to calculate the number of hospitalizations and ICU stays. The frequency of diagnosis codes is summed up for each location each day. The dataset has records for a total of 453,089 patients across the entire timespan of the JHU dataset. There are a total of 48 unique ICD-10 codes related to COVID-19 that were claimed from the set of codes considered, as shown in **Table 1**. Except the codes for defining COVID-19, we also include the codes of influenza and pneumonias because they are important symptoms of COVID-19. Even those features are not directly related to COVID-19, deep learning models are good at processing high-dimensional features and determine the proper weights assigning to those features. Hence, including those codes do not harm the model performance but may help the model to capture useful patterns from potential misdiagnosis and co-infections. Detailed county-level and state-level statistics are shown in **Table 2** and **Table 3**.

**Table 1.** ICD-10 (The 10th revision of the International statistical Classification of Diseases) codes used in our dataset. We choose codes that are relevant to the COVID-19 symptoms.

| ICD-10 | Description |
| --- | --- |
| R05 | Cough |
| R0602 | Shortness of breath |
| R509 | Fever, unspecified |
| U071 | COVID-19, virus identified |
| Z03818 | Encounter for observation for suspected exposure to other biological agents ruled out |
| Z20828 | Contact with and (suspected) exposure to other viral communicable diseases |
| B342 | Coronavirus infection, unspecified |
| B9729 | Other coronavirus as the cause of diseases classified elsewhere |
| J09 | Influenza due to certain identified influenza viruses |
| J10 | Influenza due to other identified influenza virus |
| J100 | Influenza due to other identified influenza virus with pneumonia |
| J101 | Influenza due to other identified influenza virus with other respiratory manifestations |
| J108 | Influenza due to other identified influenza virus with other manifestations |
| J11 | Influenza due to unidentified influenza virus |
| J110 | Influenza due to unidentified influenza virus with pneumonia |
| J111 | Influenza due to unidentified influenza virus with other respiratory manifestations |
| J118 | Influenza due to unidentified influenza virus with other manifestations |
| J12 | Viral pneumonia, not elsewhere classified |
| J120 | Adenoviral pneumonia |
| J1289 | Other viral pneumonia |
| J13 | Pneumonia due to Streptococcus pneumoniae |
| J14 | Pneumonia due to Hemophilus influenzae |
| J15 | Bacterial pneumonia, not elsewhere classified |
| J150 | Pneumonia due to Klebsiella pneumoniae |
| J151 | Pneumonia due to Pseudomonas |
| J152 | Pneumonia due to staphylococcus |
| J153 | Pneumonia due to streptococcus, group B |
| J154 | Pneumonia due to other streptococci |
| J155 | Pneumonia due to Escherichia coli |
| J156 | Pneumonia due to other Gram-negative bacteria |
| J157 | Pneumonia due to Mycoplasma pneumoniae |
| J158 | Pneumonia due to other specified bacteria |
| J159 | Unspecified bacterial pneumonia |
| J16 | Pneumonia due to other infectious organisms, not elsewhere classified |
| J160 | Chlamydial pneumonia |
| J168 | Pneumonia due to other specified infectious organisms |
| J17 | Pneumonia in diseases classified elsewhere |
| J18 | Pneumonia, unspecified organism |
| J180 | Bronchopneumonia, unspecified organism |
| J181 | Lobar pneumonia, unspecified organism |
| J182 | Hypostatic pneumonia, unspecified organism |
| J188 | Other pneumonia, unspecified organism |
| J189 | Pneumonia, unspecified organism |
| J208 | Acute bronchitis due to other specified organisms |
| J22 | Unspecified acute lower respiratory infection |
| J40 | Bronchitis, not specified as acute or chronic |
| J80 | Acute respiratory distress syndrome |
| J988 | Other specified respiratory disorders |

**Table 2.** County-level statistics

| County | Patient # | Bed # | Site # | Visit # | Avg code # | Male % | Avg male age | Avg female age |
| --- | --- | --- | --- | --- | --- | --- | --- | --- |
| AL_JEFFERSON | 314758 | 4965 | 17 | 308 | 367 | 0.38 | 57 | 58 |
| AL_MOBILE | 136442 | 1983 | 8 | 274 | 369 | 0.42 | 61 | 63 |
| AZ_APACHE | 4494 | 160 | 4 | 2 | 4 | 0.41 | 51 | 52 |
| AZ_MARICOPA | 890970 | 11891 | 69 | 1385 | 1744 | 0.44 | 50 | 52 |
| AZ_NAVAJO | 14440 | 176 | 5 | 17 | 22 | 0.45 | 50 | 52 |
| AZ_PIMA | 176903 | 3566 | 20 | 187 | 234 | 0.44 | 53 | 53 |
| CA_ALAMEDA | 178694 | 3927 | 22 | 110 | 130 | 0.46 | 53 | 55 |
| CA_CONTRA COSTA | 202720 | 1739 | 9 | 167 | 197 | 0.41 | 56 | 58 |
| CA_FRESNO | 119063 | 3262 | 14 | 114 | 131 | 0.43 | 49 | 51 |
| CA_KERN | 161935 | 1890 | 15 | 155 | 184 | 0.4 | 53 | 57 |
| CA_LOS ANGELES | 1916739 | 28427 | 120 | 1006 | 1193 | 0.43 | 54 | 56 |
| CA_ORANGE | 825228 | 6490 | 34 | 589 | 671 | 0.45 | 57 | 58 |
| CA_RIVERSIDE | 270597 | 4284 | 25 | 153 | 181 | 0.43 | 59 | 60 |
| CA_SACRAMENTO | 380041 | 3274 | 17 | 172 | 202 | 0.42 | 53 | 55 |
| CA_SAN BERNARDINO | 268993 | 4967 | 30 | 123 | 142 | 0.42 | 56 | 57 |
| CA_SAN DIEGO | 408797 | 7997 | 32 | 263 | 318 | 0.41 | 56 | 58 |
| CA_SAN FRANCISCO | 76289 | 2676 | 13 | 47 | 56 | 0.41 | 53 | 55 |
| CA_SAN MATEO | 93222 | 2311 | 10 | 36 | 43 | 0.45 | 56 | 57 |
| CA_SANTA BARBARA | 69333 | 1151 | 8 | 40 | 48 | 0.43 | 54 | 57 |
| CA_SANTA CLARA | 175973 | 4831 | 17 | 88 | 104 | 0.43 | 48 | 47 |
| CA_TULARE | 98385 | 1502 | 6 | 60 | 67 | 0.42 | 48 | 49 |
| CO_ADAMS | 93915 | 1587 | 8 | 93 | 126 | 0.45 | 41 | 40 |
| CO_ARAPAHOE | 182502 | 1356 | 9 | 236 | 288 | 0.43 | 53 | 53 |
| CO_DENVER | 144436 | 2946 | 14 | 161 | 195 | 0.44 | 50 | 51 |
| CO_EL PASO | 83087 | 1833 | 11 | 70 | 87 | 0.45 | 46 | 41 |
| CO_JEFFERSON | 175778 | 748 | 5 | 238 | 289 | 0.46 | 55 | 54 |
| CO_WELD | 23798 | 598 | 5 | 17 | 23 | 0.45 | 51 | 50 |
| CT_FAIRFIELD | 187131 | 2428 | 9 | 214 | 282 | 0.43 | 53 | 53 |
| CT_HARTFORD | 226979 | 2271 | 13 | 241 | 320 | 0.43 | 57 | 59 |
| CT_LITCHFIELD | 28116 | 272 | 3 | 23 | 30 | 0.44 | 58 | 57 |
| CT_NEW HAVEN | 157756 | 3390 | 14 | 336 | 458 | 0.44 | 57 | 58 |
| DC_DISTRICT OF COLUMBIA | 226327 | 3510 | 12 | 260 | 336 | 0.41 | 47 | 49 |
| DE_KENT | 36709 | 400 | 3 | 47 | 58 | 0.41 | 48 | 52 |
| DE_NEW CASTLE | 193503 | 1409 | 10 | 176 | 234 | 0.44 | 50 | 49 |
| DE_SUSSEX | 60198 | 571 | 4 | 80 | 102 | 0.42 | 55 | 57 |
| FL_BROWARD | 455694 | 6711 | 26 | 654 | 825 | 0.44 | 53 | 53 |
| FL_COLLIER | 90050 | 1018 | 6 | 67 | 81 | 0.45 | 62 | 64 |
| FL_DUVAL | 341776 | 4198 | 15 | 275 | 340 | 0.44 | 54 | 55 |
| FL_HILLSBOROUGH | 685552 | 5022 | 22 | 451 | 542 | 0.43 | 59 | 61 |
| FL_LEE | 203463 | 1850 | 9 | 149 | 178 | 0.43 | 59 | 62 |
| FL_MIAMI-DADE | 693745 | 10704 | 40 | 812 | 1056 | 0.44 | 55 | 55 |
| FL_ORANGE | 358461 | 4627 | 23 | 429 | 528 | 0.43 | 51 | 52 |
| FL_PALM BEACH | 322627 | 4958 | 24 | 510 | 654 | 0.43 | 58 | 58 |
| FL_PINELLAS | 213141 | 4370 | 18 | 188 | 217 | 0.45 | 62 | 62 |
| GA_COBB | 140826 | 1442 | 5 | 173 | 221 | 0.41 | 54 | 56 |
| GA_DEKALB | 128609 | 2737 | 11 | 120 | 149 | 0.41 | 49 | 50 |
| GA_DOUGHERTY | 23680 | 698 | 2 | 17 | 25 | 0.38 | 48 | 55 |
| GA_FULTON | 292492 | 5165 | 16 | 317 | 406 | 0.41 | 52 | 55 |
| GA_GWINNETT | 162247 | 975 | 7 | 148 | 183 | 0.42 | 51 | 50 |
| GA_HALL | 65940 | 557 | 1 | 77 | 93 | 0.43 | 53 | 56 |
| IA_BLACK HAWK | 27536 | 671 | 3 | 29 | 36 | 0.44 | 53 | 56 |
| IA_POLK | 153287 | 2850 | 11 | 135 | 165 | 0.43 | 48 | 49 |
| IA_WOODBURY | 32998 | 584 | 2 | 44 | 52 | 0.45 | 47 | 49 |
| IL_COOK | 1047503 | 20995 | 80 | 1338 | 1852 | 0.43 | 49 | 51 |
| IL_DUPAGE | 326598 | 2454 | 11 | 403 | 581 | 0.43 | 54 | 55 |
| IL_KANE | 88889 | 1280 | 5 | 72 | 94 | 0.43 | 49 | 51 |
| IL_LAKE | 197634 | 1818 | 8 | 195 | 273 | 0.41 | 53 | 53 |
| IL_MCHENRY | 53933 | 468 | 4 | 63 | 86 | 0.44 | 49 | 50 |
| IL_WILL | 131192 | 1003 | 4 | 144 | 217 | 0.44 | 53 | 52 |
| IL_WINNEBAGO | 56610 | 1336 | 6 | 50 | 62 | 0.45 | 49 | 52 |
| IN_ALLEN | 154365 | 2062 | 12 | 173 | 256 | 0.43 | 55 | 58 |
| IN_CASS | 10605 | 268 | 3 | 16 | 27 | 0.42 | 53 | 55 |
| IN_LAKE | 90733 | 3102 | 15 | 221 | 321 | 0.44 | 55 | 57 |
| IN_MARION | 205269 | 5555 | 29 | 280 | 357 | 0.41 | 57 | 57 |
| IN_ST. JOSEPH | 69136 | 1045 | 8 | 81 | 106 | 0.43 | 54 | 56 |
| KS_FINNEY | 6000 | 102 | 1 | 1 | 1 | 0.45 | 46 | 44 |
| KS_FORD | 6740 | 99 | 1 | 11 | 14 | 0.43 | 49 | 52 |
| KS_LEAVENWORTH | 8386 | 429 | 3 | 6 | 7 | 0.47 | 60 | 61 |
| KS_WYANDOTTE | 21954 | 546 | 5 | 17 | 20 | 0.43 | 54 | 53 |
| KY_JEFFERSON | 283697 | 4707 | 17 | 364 | 464 | 0.42 | 52 | 54 |
| MA_BARNSTABLE | 46632 | 436 | 4 | 46 | 58 | 0.47 | 59 | 58 |
| MA_BRISTOL | 121026 | 1535 | 10 | 165 | 199 | 0.44 | 56 | 55 |
| MA_ESSEX | 193627 | 1645 | 14 | 273 | 376 | 0.41 | 55 | 56 |
| MA_HAMPDEN | 108738 | 1882 | 12 | 224 | 300 | 0.44 | 57 | 57 |
| MA_MIDDLESEX | 457272 | 4349 | 23 | 520 | 692 | 0.44 | 55 | 56 |
| MA_NORFOLK | 135880 | 1426 | 10 | 153 | 195 | 0.41 | 50 | 50 |
| MA_PLYMOUTH | 67009 | 1385 | 8 | 71 | 87 | 0.44 | 53 | 52 |
| MA_SUFFOLK | 188818 | 8339 | 24 | 323 | 487 | 0.44 | 52 | 53 |
| MA_WORCESTER | 113729 | 2313 | 16 | 163 | 203 | 0.45 | 55 | 54 |
| MD_ANNE ARUNDEL | 156853 | 629 | 2 | 179 | 234 | 0.43 | 50 | 53 |
| MD_BALTIMORE | 399555 | 6557 | 24 | 440 | 555 | 0.43 | 53 | 54 |
| MD_FREDERICK | 80759 | 269 | 1 | 80 | 116 | 0.45 | 53 | 55 |
| MD_HOWARD | 78631 | 576 | 3 | 100 | 127 | 0.42 | 51 | 53 |
| MD_MONTGOMERY | 278721 | 1776 | 10 | 366 | 466 | 0.42 | 48 | 50 |
| MD_PRINCE GEORGE'S | 212207 | 1057 | 7 | 233 | 298 | 0.41 | 52 | 53 |
| MI_GENESEE | 138912 | 1210 | 4 | 154 | 210 | 0.43 | 56 | 58 |
| MI_KENT | 365754 | 2319 | 12 | 269 | 327 | 0.42 | 50 | 53 |
| MI_MACOMB | 206173 | 1414 | 8 | 497 | 726 | 0.44 | 57 | 59 |
| MI_OAKLAND | 400174 | 4980 | 19 | 826 | 1190 | 0.44 | 56 | 57 |
| MI_WASHTENAW | 255637 | 2165 | 9 | 344 | 558 | 0.44 | 52 | 55 |
| MI_WAYNE | 415927 | 6963 | 30 | 828 | 1193 | 0.43 | 53 | 54 |
| MN_HENNEPIN | 451162 | 5751 | 17 | 337 | 451 | 0.43 | 51 | 54 |
| MN_NOBLES | 5990 | 48 | 1 | 6 | 9 | 0.47 | 51 | 54 |
| MN_RAMSEY | 205877 | 1885 | 7 | 83 | 100 | 0.41 | 49 | 51 |
| MN_STEARNS | 25176 | 569 | 4 | 7 | 8 | 0.46 | 53 | 58 |
| MO_ST. LOUIS | 314423 | 8401 | 34 | 249 | 317 | 0.42 | 55 | 56 |
| NC_DURHAM | 42238 | 1665 | 6 | 22 | 26 | 0.34 | 56 | 60 |
| NC_MECKLENBURG | 277203 | 3166 | 17 | 160 | 202 | 0.43 | 51 | 51 |
| NC_WAKE | 192235 | 2231 | 13 | 152 | 179 | 0.44 | 49 | 47 |
| ND_CASS | 24035 | 1300 | 9 | 15 | 19 | 0.45 | 49 | 51 |
| NE_DAKOTA | 4916 | 0 | 0 | 3 | 3 | 0.45 | 50 | 52 |
| NE_DOUGLAS | 133376 | 2995 | 19 | 97 | 122 | 0.43 | 51 | 53 |
| NE_HALL | 31979 | 159 | 1 | 44 | 61 | 0.42 | 51 | 53 |
| NH_HILLSBOROUGH | 81543 | 1110 | 8 | 88 | 116 | 0.44 | 52 | 53 |
| NH_ROCKINGHAM | 78225 | 601 | 6 | 126 | 152 | 0.44 | 56 | 57 |
| NJ_ATLANTIC | 57671 | 1567 | 6 | 62 | 77 | 0.44 | 54 | 55 |
| NJ_BERGEN | 570476 | 4410 | 14 | 833 | 1217 | 0.41 | 52 | 55 |
| NJ_BURLINGTON | 115186 | 1155 | 9 | 125 | 176 | 0.43 | 55 | 57 |
| NJ_CAMDEN | 172336 | 2625 | 9 | 277 | 383 | 0.43 | 56 | 55 |
| NJ_CUMBERLAND | 30538 | 362 | 3 | 42 | 55 | 0.45 | 53 | 54 |
| NJ_ESSEX | 265006 | 4210 | 13 | 456 | 653 | 0.41 | 52 | 53 |
| NJ_GLOUCESTER | 58628 | 515 | 2 | 53 | 67 | 0.42 | 49 | 51 |
| NJ_HUDSON | 86105 | 1812 | 7 | 250 | 354 | 0.42 | 53 | 53 |
| NJ_MERCER | 288422 | 1853 | 9 | 195 | 249 | 0.28 | 45 | 54 |
| NJ_MIDDLESEX | 203129 | 2704 | 11 | 607 | 852 | 0.43 | 54 | 55 |
| NJ_MONMOUTH | 168254 | 2681 | 8 | 481 | 783 | 0.44 | 53 | 53 |
| NJ_MORRIS | 149111 | 2113 | 10 | 408 | 662 | 0.45 | 53 | 54 |
| NJ_OCEAN | 136542 | 1707 | 9 | 213 | 271 | 0.47 | 54 | 53 |
| NJ_PASSAIC | 137475 | 1187 | 5 | 304 | 454 | 0.43 | 51 | 52 |
| NJ_SOMERSET | 291752 | 642 | 2 | 255 | 365 | 0.4 | 47 | 51 |
| NJ_UNION | 161568 | 2179 | 10 | 511 | 708 | 0.43 | 51 | 51 |
| NM_BERNALILLO | 111409 | 2838 | 18 | 107 | 129 | 0.42 | 54 | 55 |
| NM_MCKINLEY | 3826 | 194 | 4 | 3 | 5 | 0.44 | 62 | 62 |
| NM_SAN JUAN | 17311 | 270 | 3 | 28 | 31 | 0.44 | 58 | 59 |
| NV_CLARK | 498324 | 6825 | 45 | 668 | 877 | 0.43 | 56 | 57 |
| NV_WASHOE | 83221 | 1965 | 13 | 100 | 130 | 0.44 | 54 | 55 |
| NY_ALBANY | 165355 | 1627 | 7 | 221 | 281 | 0.43 | 53 | 55 |
| NY_DUTCHESS | 118691 | 976 | 5 | 120 | 144 | 0.43 | 52 | 53 |
| NY_ERIE | 224088 | 3499 | 15 | 253 | 324 | 0.43 | 55 | 56 |
| NY_MONROE | 134568 | 1597 | 5 | 69 | 84 | 0.41 | 58 | 60 |
| NY_NASSAU | 670656 | 5045 | 16 | 1826 | 2749 | 0.44 | 51 | 52 |
| NY_NEW YORK | 1112742 | 10863 | 28 | 2450 | 3597 | 0.41 | 52 | 53 |
| NY_ONONDAGA | 147345 | 2031 | 7 | 125 | 149 | 0.42 | 54 | 56 |
| NY_ORANGE | 100906 | 1137 | 6 | 369 | 489 | 0.43 | 51 | 52 |
| NY_PUTNAM | 24692 | 347 | 2 | 34 | 44 | 0.4 | 54 | 54 |
| NY_ROCKLAND | 108328 | 1293 | 5 | 319 | 461 | 0.44 | 53 | 54 |
| NY_SUFFOLK | 379923 | 4718 | 16 | 1007 | 1468 | 0.43 | 51 | 52 |
| NY_SULLIVAN | 9473 | 174 | 2 | 14 | 19 | 0.44 | 52 | 54 |
| NY_ULSTER | 41779 | 175 | 2 | 63 | 83 | 0.44 | 57 | 57 |
| NY_WESTCHESTER | 339702 | 4401 | 21 | 755 | 1062 | 0.42 | 52 | 53 |
| OH_CUYAHOGA | 373592 | 7501 | 32 | 365 | 454 | 0.42 | 54 | 56 |
| OH_FRANKLIN | 570600 | 6918 | 31 | 376 | 459 | 0.41 | 47 | 49 |
| OH_HAMILTON | 415904 | 4922 | 23 | 382 | 478 | 0.43 | 46 | 45 |
| OH_LUCAS | 213970 | 3351 | 17 | 198 | 269 | 0.42 | 52 | 54 |
| OH_MAHONING | 70179 | 1147 | 9 | 170 | 225 | 0.43 | 55 | 56 |
| OH_MARION | 21146 | 270 | 1 | 15 | 18 | 0.42 | 49 | 52 |
| OH_PICKAWAY | 11713 | 94 | 1 | 5 | 6 | 0.42 | 53 | 56 |
| OK_OKLAHOMA | 202583 | 5371 | 42 | 181 | 220 | 0.42 | 56 | 57 |
| PA_ALLEGHENY | 356957 | 7765 | 34 | 295 | 351 | 0.43 | 57 | 58 |
| PA_BERKS | 75509 | 1399 | 7 | 85 | 114 | 0.43 | 54 | 58 |
| PA_BUCKS | 208543 | 1343 | 9 | 244 | 345 | 0.42 | 56 | 57 |
| PA_CHESTER | 181856 | 1011 | 7 | 134 | 168 | 0.42 | 53 | 54 |
| PA_DELAWARE | 148302 | 1668 | 9 | 143 | 192 | 0.42 | 54 | 54 |
| PA_LACKAWANNA | 52668 | 1042 | 6 | 68 | 86 | 0.44 | 58 | 59 |
| PA_LANCASTER | 100228 | 1093 | 6 | 71 | 96 | 0.43 | 50 | 51 |
| PA_LEHIGH | 111238 | 1372 | 9 | 182 | 246 | 0.4 | 50 | 52 |
| PA_LUZERNE | 61895 | 501 | 6 | 50 | 62 | 0.42 | 58 | 59 |
| PA_MONROE | 30662 | 345 | 2 | 45 | 62 | 0.42 | 51 | 53 |
| PA_MONTGOMERY | 418518 | 3207 | 16 | 339 | 458 | 0.4 | 57 | 59 |
| PA_NORTHAMPTON | 91598 | 1171 | 6 | 130 | 177 | 0.43 | 49 | 52 |
| PA_PHILADELPHIA | 344652 | 8747 | 36 | 629 | 891 | 0.43 | 49 | 51 |
| RI_PROVIDENCE | 251120 | 3010 | 13 | 358 | 424 | 0.43 | 52 | 53 |
| SC_GREENVILLE | 170969 | 1793 | 15 | 138 | 175 | 0.43 | 51 | 51 |
| SC_RICHLAND | 138916 | 2545 | 13 | 95 | 113 | 0.42 | 50 | 50 |
| SD_MINNEHAHA | 78776 | 1487 | 10 | 137 | 170 | 0.43 | 56 | 59 |
| TN_DAVIDSON | 375090 | 3570 | 16 | 208 | 257 | 0.4 | 51 | 54 |
| TN_SHELBY | 319585 | 5388 | 26 | 474 | 620 | 0.4 | 52 | 55 |
| TN_TROUSDALE | 568 | 25 | 1 | 0 | 0 | 0.41 | 58 | 53 |
| TX_BEXAR | 328337 | 8432 | 46 | 321 | 367 | 0.43 | 54 | 54 |
| TX_COLLIN | 302636 | 2446 | 21 | 237 | 290 | 0.4 | 53 | 53 |
| TX_DALLAS | 716189 | 9899 | 56 | 753 | 953 | 0.38 | 51 | 54 |
| TX_DENTON | 173703 | 1638 | 19 | 144 | 179 | 0.43 | 52 | 53 |
| TX_EL PASO | 97053 | 3043 | 20 | 123 | 140 | 0.44 | 56 | 56 |
| TX_FORT BEND | 97058 | 1380 | 13 | 138 | 180 | 0.42 | 51 | 53 |
| TX_HARRIS | 758706 | 17394 | 95 | 995 | 1264 | 0.43 | 47 | 48 |
| TX_POTTER | 68856 | 1403 | 9 | 146 | 172 | 0.43 | 57 | 58 |
| TX_TARRANT | 361685 | 6122 | 44 | 514 | 656 | 0.42 | 53 | 55 |
| TX_TRAVIS | 694270 | 3367 | 23 | 633 | 801 | 0.39 | 49 | 52 |
| UT_SALT LAKE | 162528 | 2451 | 19 | 143 | 176 | 0.43 | 51 | 51 |
| UT_UTAH | 63606 | 1353 | 10 | 44 | 55 | 0.45 | 47 | 46 |
| VA_ARLINGTON | 55046 | 370 | 1 | 63 | 89 | 0.41 | 49 | 51 |
| VA_CHESTERFIELD | 97216 | 490 | 3 | 44 | 51 | 0.42 | 51 | 51 |
| VA_FAIRFAX | 368091 | 2013 | 8 | 335 | 455 | 0.42 | 49 | 49 |
| VA_HENRICO | 194873 | 704 | 4 | 155 | 183 | 0.42 | 50 | 47 |
| VA_LOUDOUN | 95930 | 487 | 5 | 119 | 156 | 0.44 | 49 | 49 |
| VA_PRINCE WILLIAM | 62217 | 243 | 2 | 62 | 87 | 0.42 | 48 | 49 |
| WA_KING | 369636 | 5871 | 28 | 341 | 440 | 0.42 | 50 | 52 |
| WA_PIERCE | 172742 | 2541 | 11 | 182 | 231 | 0.43 | 48 | 49 |
| WA_SNOHOMISH | 84202 | 1017 | 8 | 84 | 111 | 0.43 | 51 | 52 |
| WA_YAKIMA | 36485 | 528 | 4 | 52 | 64 | 0.44 | 53 | 55 |
| WI_BROWN | 75291 | 1278 | 8 | 73 | 94 | 0.45 | 53 | 57 |
| WI_MILWAUKEE | 179429 | 5441 | 22 | 281 | 347 | 0.42 | 50 | 53 |
| WI_RACINE | 17972 | 665 | 4 | 5 | 6 | 0.43 | 48 | 48 |

**Table 3.** State-level statistics

| State | Patient # | Bed # | Site # | Visit # | Avg code # | Male % | Avg male age | Avg female age |
| --- | --- | --- | --- | --- | --- | --- | --- | --- |
| CA | 11280624 | 95697 | 510 | 3679 | 4305 | 0.43 | 55.43 | 56.72 |
| NJ | 6318697 | 32850 | 134 | 5258 | 7573 | 0.43 | 52.67 | 54.1 |
| NY | 11672957 | 66807 | 266 | 11791 | 16587 | 0.43 | 54.77 | 56.34 |
| WV | 864456 | 8527 | 75 | 402 | 474 | 0.43 | 56.33 | 57.11 |
| VA | 4086215 | 12590 | 86 | 1205 | 1519 | 0.42 | 54.59 | 54.75 |
| MN | 2230003 | 17462 | 157 | 681 | 863 | 0.44 | 56.59 | 58.03 |
| CT | 1371913 | 10469 | 50 | 922 | 1226 | 0.44 | 56.75 | 57.5 |
| GA | 4672277 | 30709 | 196 | 2341 | 2922 | 0.4 | 56.01 | 56.44 |
| WI | 2163502 | 19661 | 160 | 785 | 994 | 0.44 | 54.44 | 56.93 |
| IA | 1541463 | 12487 | 130 | 661 | 844 | 0.44 | 52.27 | 53.39 |
| AR | 1142575 | 12755 | 111 | 642 | 779 | 0.44 | 58 | 57.93 |
| OK | 1505373 | 16185 | 175 | 804 | 997 | 0.43 | 59.47 | 59.69 |
| NC | 4393806 | 29450 | 180 | 1586 | 1905 | 0.42 | 56.8 | 56.56 |
| OR | 1736670 | 10202 | 77 | 615 | 779 | 0.43 | 55.19 | 56.89 |
| TX | 12470003 | 93267 | 719 | 6318 | 7796 | 0.43 | 55.52 | 56.05 |
| ND | 286099 | 3782 | 57 | 118 | 148 | 0.44 | 52.33 | 53.25 |
| OH | 7160485 | 46531 | 282 | 3230 | 4012 | 0.43 | 52.28 | 53.92 |
| SD | 468914 | 4499 | 72 | 252 | 310 | 0.44 | 59.11 | 59.95 |
| ID | 603338 | 4199 | 60 | 263 | 318 | 0.44 | 55.42 | 57.33 |
| CO | 2340901 | 15125 | 124 | 1183 | 1474 | 0.44 | 53.48 | 53.89 |
| NE | 848369 | 7791 | 107 | 344 | 439 | 0.45 | 53.93 | 55.49 |
| KY | 2869138 | 18306 | 130 | 1474 | 1828 | 0.42 | 51.74 | 51.52 |
| FL | 10673839 | 71431 | 364 | 5620 | 6899 | 0.44 | 59.91 | 60.88 |
| KS | 1245960 | 13062 | 172 | 445 | 553 | 0.44 | 53.5 | 54.22 |
| UT | 918481 | 5914 | 64 | 341 | 419 | 0.46 | 50.79 | 50.93 |
| AL | 2307017 | 20490 | 132 | 1534 | 1924 | 0.43 | 59.61 | 59.49 |
| ME | 749670 | 4533 | 43 | 336 | 416 | 0.43 | 57.69 | 60.81 |
| IL | 5313832 | 39999 | 229 | 2887 | 3966 | 0.43 | 52.61 | 53.69 |
| NM | 605791 | 5587 | 58 | 217 | 255 | 0.43 | 52.73 | 53.39 |
| PA | 6351803 | 46230 | 281 | 3273 | 4297 | 0.43 | 56.01 | 57.15 |
| TN | 4199028 | 25133 | 169 | 2021 | 2504 | 0.43 | 56.91 | 56.53 |
| WA | 2661115 | 17129 | 127 | 1222 | 1558 | 0.43 | 48.9 | 50.92 |
| IN | 3055544 | 23641 | 198 | 1536 | 2034 | 0.43 | 56.92 | 57.8 |
| MO | 1932287 | 23007 | 164 | 850 | 1064 | 0.43 | 56.96 | 57.39 |
| MI | 5543648 | 30763 | 190 | 3764 | 5276 | 0.44 | 56.27 | 58.5 |
| SC | 2277366 | 14817 | 104 | 1144 | 1424 | 0.41 | 53.91 | 54.46 |
| NH | 547482 | 3101 | 33 | 306 | 385 | 0.44 | 56.5 | 58.2 |
| MS | 1247700 | 16605 | 130 | 749 | 925 | 0.41 | 56.07 | 55.89 |
| AZ | 2987234 | 18730 | 133 | 1847 | 2318 | 0.43 | 51.33 | 52.33 |
| MA | 2978862 | 24108 | 130 | 2018 | 2698 | 0.44 | 53.57 | 54.43 |
| NV | 1178120 | 9380 | 73 | 817 | 1069 | 0.46 | 55 | 56.38 |
| RI | 783321 | 3723 | 17 | 552 | 659 | 0.44 | 53.6 | 53.2 |
| MD | 3541594 | 13848 | 71 | 1908 | 2434 | 0.43 | 51.48 | 53.43 |
| DE | 577730 | 2380 | 17 | 303 | 395 | 0.42 | 51 | 52.67 |
| DC | 383908 | 3510 | 12 | 260 | 336 | 0.41 | 47 | 49 |

## IMPLEMENTATION DETAILS

All methods are implemented in PyTorch 1.1 and trained on a server equipped with an Intel Xeon E5-2620 Octa-Core CPU, 256GB Memory and a Titan V GPU. For the hyper-parameters of baseline models, we follow the recommended setting if it is available in the original paper. Otherwise, we determine its value by grid search on the validation set.

For the STAN model, the hidden dimension of the GRU is set to 200 and the hidden dimension of the MLP is set to 100. The graph embedding dimension is set to 400 and the graph attention dimension is set to 650. The input sliding window $L_{I}$ is set to 5. The $r$ is set to 30, $\alpha$ and $\beta$ is set to 0.35 and 0.37.

For the GRU model, the hidden dimension is set to 100. For the ColaGNN, the hidden dimension of GRU is set to 256, the dimension of graph node embedding is set to 500. For the CovidGNN model, we use a two-layer GNN and the dimension of graph node embedding is set to 256.

## PERFORMANCE DETAILS

We report the prediction MSE for each state and county in **Table 2** and **Table 3**. Due to space limits, we only take SIR and SEIR into comparison. For all 45 states, when $L_{P}=5$, STAN achieves the best performance on 37 states; when $L_{P}=15 and 20$, STAN achieves the best performance on 35 states. For all 45 states, when $L_{P}=5$, STAN achieves the best performance on 37 states; when $L_{P}=15 and 20$, STAN achieves the best performance on 35 states.

For all 193 counties, when $L_{P}=5$, STAN achieves the best performance on 134 counties; when $L_{P}=15$, STAN achieves the best performance on 148 states; when $L_{P}=20$, STAN achieves the best performance on 143 states.

While conducting county-level prediction, STAN can achieve better long-term prediction compared to SIR and SEIR on most locations. This is due to the location graph is more granular and the model can extract detailed spatial interactions between nodes. And also for some locations, the pandemic haven’t outbreak, so STAN can better predict future progression by considering progressions from neighboring locations. While aggregating the data and conducting state-level predictions, STAN’s performance is more consistent over all length of prediction window.

**Table 4.** State-wise prediction MSE

| State | $\boldsymbol{L}_{\boldsymbol{P}}\boldsymbol{=5}$ | | | $\boldsymbol{L}_{\boldsymbol{P}}\boldsymbol{=15}$ | | | $\boldsymbol{L}_{\boldsymbol{P}}\boldsymbol{=20}$ | | |
| --- | --- | --- | --- | --- | --- | --- | --- | --- | --- |
|  | SIR | SEIR | STAN | SIR | SEIR | STAN | SIR | SEIR | STAN |
| AL | 35969 | **33840** | 50841 | **1148638** | 1325467 | 2143502 | **2245236** | 2717692 | 2934974 |
| AR | 525720 | 284850 | **17204** | 3725935 | 1829427 | **863864** | 7632371 | 3919630 | **1223407** |
| AZ | 47235 | 116290 | **43536** | 213314 | 771706 | **29750** | 2036012 | 4029891 | **248002** |
| CA | 2822766 | **638352** | 1781510 | 58355218 | 19821426 | **788942** | 156482909 | 58379735 | **2782909** |
| CO | 573072 | 130782 | **829** | 6361723 | 2275589 | **12394** | 14264220 | 5944874 | **422423** |
| CT | 5081367 | 1917780 | **141097** | 19189818 | 3110738 | **717895** | 33225807 | 4117263 | **1538260** |
| DC | **3221** | 31441 | 18307 | **12073** | 507313 | 61560 | 44444 | 1060394 | **31670** |
| DE | 216513 | 19855 | **5568** | 1173115 | **11306** | 36567 | 2134180 | **25150** | 84301 |
| FL | 1333562 | 770617 | **156373** | 8958648 | 3979007 | **3735569** | 19716893 | 8349910 | **5438116** |
| GA | 478806 | **40364** | 188259 | 5150993 | 8394772 | **2572506** | 7531756 | 6422158 | **529251** |
| IA | 790491 | 341599 | **9753** | 11584529 | 4250450 | **499438** | 23847090 | 8362259 | **1243471** |
| ID | 3968 | **2679** | 2709 | 24947 | **13793** | 14486 | 53712 | **28624** | 30156 |
| IL | 7677558 | 4539416 | **787052** | 87050468 | 47549378 | **11871478** | 174425565 | **90124497** | 102453656 |
| IN | 998649 | 1207340 | **14134** | 9804565 | 9782019 | **77851** | 22376904 | 20784999 | **99796** |
| KS | 105276 | 31842 | **25305** | 1856691 | 771430 | **131930** | 4089669 | 1838994 | **712897** |
| KY | 49469 | 50893 | **9794** | 275923 | 253590 | **174215** | 987554 | 886238 | **40289** |
| MA | 34638912 | 3430794 | **49765** | 188760053 | 10321814 | **694645** | 376509655 | 28495989 | **2771604** |
| MD | 2783292 | 3147157 | **82780** | 5010187 | 4771543 | **840472** | 17082240 | 15415005 | **1418938** |
| ME | 6411 | **1054** | 3201 | 40369 | **17499** | 28308 | 58277 | **27665** | 68965 |
| MI | 5163810 | 1825336 | **29887** | 4247109 | 1762722 | **228764** | 12098923 | 1337059 | **661681** |
| MN | 88407 | 1456259 | **2704** | **3197951** | 6891496 | 4023834 | **8432004** | 10666408 | 18165335 |
| MO | 23671 | 132373 | **1097** | 4317008 | 2754554 | **4925** | 3442633 | 2130238 | **7627** |
| MS | 98457 | 83079 | **21409** | 1661032 | 1445189 | **133923** | 4058397 | 3568215 | **80754** |
| NC | 642968 | 388320 | **68220** | 8984167 | 4927417 | **4438426** | 21713680 | 11804975 | **7397819** |
| ND | 33225 | 10393 | **7156** | 223308 | 48529 | **36832** | 333595 | 45467 | **41254** |
| NE | 1797250 | 452137 | **15271** | 12913421 | 2482806 | **134415** | 24967634 | 5231907 | **1761118** |
| NH | 7540 | 14549 | **5795** | 122793 | 187446 | **10922** | 286786 | 405453 | **15569** |
| NJ | 21547538 | 11697626 | **1364570** | 165123676 | 102546164 | **430633** | 328017796 | 218066841 | **1153618** |
| NM | 101140 | 20694 | **2188** | 248806 | 39101 | **2572** | 742942 | 78696 | **56863** |
| NV | 114262 | 47199 | **27649** | 768725 | 234978 | **126553** | 1490263 | 409414 | **5774** |
| NY | 41029629 | 32073349 | **1439871** | 344142511 | 276121074 | **265175** | 705405848 | 572633149 | **62852285** |
| OH | 99736 | 70303 | **33923** | **154392** | 2612732 | 239138 | **212192** | 6303436 | 704602 |
| OK | 18380 | 24159 | **1264** | 80102 | 118184 | **13805** | 134715 | 205189 | **26168** |
| OR | 1604 | 2754 | **603** | 29013 | 28568 | **23733** | 37285 | 32196 | **10637** |
| PA | 3487833 | 342445 | **274261** | 32691226 | 4210910 | **976499** | 65992492 | **9148289** | 10026111 |
| RI | 626385 | **59254** | 116616 | 1940867 | **497888** | 1862138 | 3743923 | **373901** | 2130920 |
| SC | 205482 | 70740 | **5185** | 1888462 | 555102 | **157086** | 4486233 | 1418521 | **484925** |
| SD | 28376 | 21351 | **1600** | 105991 | 59664 | **32060** | 170071 | 83093 | **2859** |
| TN | 556642 | 549156 | **90940** | 6200060 | 5601928 | **1392780** | 15920345 | 14173437 | **3181705** |
| TX | 861329 | 849580 | **105879** | 1025252 | **836880** | 2030543 | 5769692 | 4537729 | **980812** |
| UT | 72980 | 42764 | **3107** | 141176 | 95690 | **73101** | 626564 | 344090 | **161141** |
| VA | 1496684 | 519132 | **97442** | 19820177 | 32076705 | **1532963** | 15491286 | 26336937 | **3347044** |
| WA | **10767** | 69474 | 107561 | **51859** | 840258 | 356025 | **300588** | 2743506 | 1450136 |
| WI | 242847 | 420722 | **18915** | 4281439 | 5991377 | **166584** | 9248112 | 12308700 | **499113** |
| WV | 1435 | 513 | **291** | 55758 | 31789 | **24997** | 103921 | 55455 | **13567** |

**Table 5.** County-wise prediction MSE

| County | $\boldsymbol{L}_{\boldsymbol{P}}\boldsymbol{=5}$ | | | $\boldsymbol{L}_{\boldsymbol{P}}\boldsymbol{=15}$ | | | $\boldsymbol{L}_{\boldsymbol{P}}\boldsymbol{=20}$ | | |
| --- | --- | --- | --- | --- | --- | --- | --- | --- | --- |
|  | SIR | SEIR | STAN | SIR | SEIR | STAN | SIR | SEIR | STAN |
| AL_JEFFERSON | 3223 | 3574 | **2361** | 25026 | 23800 | **20589** | 42780 | 38176 | **36682** |
| AL_MOBILE | 5752 | 2438 | **505** | 80077 | 38524 | **660** | 157201 | 75622 | **746** |
| AZ_APACHE | 450 | 1029 | **442** | 1649 | 3421 | **864** | 1628 | 9840 | **859** |
| AZ_MARICOPA | 85114 | 65174 | **6480** | 674175 | 402777 | **21385** | 2582965 | 1686594 | **51516** |
| AZ_NAVAJO | 2640 | 4399 | **2606** | **11593** | 20067 | 34941 | **25471** | 41677 | 76257 |
| AZ_PIMA | 845 | 1173 | **282** | 4314 | 4797 | **608** | 29433 | 28692 | **8965** |
| CA_ALAMEDA | **740** | 1299 | 18402 | 33418 | **30643** | 36888 | 93111 | 78405 | **42128** |
| CA_CONTRA COSTA | 2802 | 3619 | **1731** | 28556 | 36088 | **11313** | 55478 | 69388 | **17466** |
| CA_FRESNO | **3067** | 5511 | 17816 | **2465** | 14245 | 51646 | **4838** | 32615 | 90271 |
| CA_KERN | **228** | 365 | 1702 | **9908** | 15342 | 21013 | **40671** | 57632 | 56686 |
| CA_LOS ANGELES | 887585 | 532537 | **72756** | 20882437 | 12642913 | **5977146** | 55618021 | 33625707 | **14844293** |
| CA_ORANGE | 9210 | 16150 | **3056** | **33995** | 122615 | 119575 | **78495** | 296715 | 230079 |
| CA_RIVERSIDE | 14398 | 19619 | **7958** | 186497 | 196933 | **106008** | 427835 | 402958 | **107403** |
| CA_SACRAMENTO | 1210 | 878 | **11** | 9750 | 6244 | **294** | 24057 | 15512 | **698** |
| CA_SAN BERNARDINO | **458** | 4293 | 4789 | **52753** | 169233 | 212855 | **112603** | 369057 | 312516 |
| CA_SAN DIEGO | 27754 | **8096** | 277704 | 282128 | **37043** | 936850 | 561704 | **42858** | 1381216 |
| CA_SAN FRANCISCO | 8611 | **611** | 9017 | 151919 | 32332 | **10709** | 294164 | 64677 | **9484** |
| CA_SAN MATEO | 2079 | 3696 | **424** | 47068 | 67371 | **9153** | 96857 | 135225 | **19984** |
| CA_SANTA BARBARA | 88832 | 17003 | **5688** | 357411 | 8953 | **8620** | 438890 | **8125** | 9160 |
| CA_SANTA CLARA | **2074** | 2707 | 3711 | 31383 | 33590 | **14573** | 56619 | 58146 | **23667** |
| CA_TULARE | 3463 | **3064** | 22501 | 68854 | **14434** | 53733 | 174311 | **23965** | 61017 |
| CO_ADAMS | 1083 | **588** | 961 | 58570 | 41413 | **1480** | 147363 | 104163 | **2291** |
| CO_ARAPAHOE | 10301 | **1800** | 6420 | 190753 | 61265 | **9943** | 406163 | 135604 | **22915** |
| CO_DENVER | 48841 | 25151 | **18333** | 731529 | 468192 | **21210** | 1375704 | 898040 | **16347** |
| CO_EL PASO | 4793 | **4787** | 12658 | 41470 | 41404 | **13538** | 51033 | 50575 | **13188** |
| CO_JEFFERSON | **107** | 752 | 3291 | **6154** | 13605 | 17224 | **8887** | 18918 | 23809 |
| CO_WELD | 209 | 2651 | **203** | 13406 | 42649 | **4979** | 32513 | 87028 | **13056** |
| CT_FAIRFIELD | **5637** | 239179 | 75862 | **51560** | 1032925 | 157826 | **40873** | 1475549 | 399440 |
| CT_HARTFORD | 208727 | **164560** | 363294 | 413916 | 326912 | **256489** | 340851 | 256428 | **206795** |
| CT_LITCHFIELD | **1080** | 3299 | 1635 | 2234 | 11614 | **1201** | **2089** | 16346 | 2888 |
| CT_NEW HAVEN | **21125** | 32971 | 44292 | 59218 | 116076 | **59152** | **49581** | 110474 | 134604 |
| DC_DISTRICT OF COLUMBIA | 42435 | **31369** | 84285 | 790182 | 642361 | **62862** | 1554373 | 1226894 | **46607** |
| DE_KENT | **66** | 448 | 70 | **400** | 1038 | 804 | 1137 | **1007** | 6249 |
| DE_NEW CASTLE | **936** | 957 | 11428 | **62516** | 65012 | 63635 | **76431** | 80919 | 78401 |
| DE_SUSSEX | **1951** | 16321 | 26268 | 93905 | 26174 | **18834** | 211874 | 49672 | **43961** |
| FL_BROWARD | 14454 | **5659** | 7503 | 229337 | 124927 | **41251** | 514592 | 297255 | **33140** |
| FL_COLLIER | **3511** | 4014 | 4995 | **50245** | 55995 | 53655 | **99613** | 112278 | 111620 |
| FL_DUVAL | 1528 | 1149 | **79** | 11303 | 8210 | **1107** | 22112 | 15954 | **4285** |
| FL_HILLSBOROUGH | 7063 | 3316 | **152** | 107204 | 57060 | **2537** | 260544 | 145734 | **15821** |
| FL_LEE | 3127 | 4147 | **980** | 29839 | 33191 | **1494** | 60561 | 63496 | **4864** |
| FL_MIAMI-DADE | 308595 | 284310 | **12765** | 915513 | 658544 | **562077** | 1553852 | 977557 | **802164** |
| FL_ORANGE | 5476 | 4007 | **3212** | 25217 | 19498 | **474** | 51890 | 41489 | **2855** |
| FL_PALM BEACH | 854 | **786** | 5374 | **3656** | 10391 | 25501 | 33870 | 58584 | **26682** |
| FL_PINELLAS | 3448 | 1982 | **31** | 9357 | 4491 | **229** | 15304 | 7335 | **521** |
| GA_COBB | 2481 | **1324** | 1439 | 69464 | 28722 | **1987** | 143476 | 61743 | **5616** |
| GA_DEKALB | 10638 | 5779 | **1669** | 237839 | 167488 | **10687** | 445521 | 312911 | **9048** |
| GA_DOUGHERTY | **38** | 813 | 2215 | 1398 | **826** | 1365 | **1657** | 1767 | 1877 |
| GA_FULTON | 1167 | 9421 | **782** | 73286 | **10496** | 49138 | 199659 | **16176** | 52948 |
| GA_GWINNETT | 17893 | 11535 | **2273** | 602187 | 474147 | **79634** | 1312633 | 1046657 | **162862** |
| GA_HALL | 38189 | 14280 | **1116** | 405607 | 218666 | **20124** | 723685 | 417116 | **38457** |
| IA_BLACK HAWK | 14356 | 29948 | **884** | 107523 | 149148 | **1617** | 195149 | 242995 | **4701** |
| IA_POLK | 189568 | 6954 | **3664** | 1813782 | 134288 | **39484** | 3189858 | 255139 | **132200** |
| IA_WOODBURY | 1922 | **1758** | 4018 | **5579** | 30735 | 15857 | 27425 | 63053 | **12815** |
| IL_COOK | 5002651 | 4072067 | **210717** | 55305862 | 41072794 | **4411589** | 108187875 | 76314647 | **16615810** |
| IL_DUPAGE | **2309** | 7809 | 5839 | **12249** | 75555 | 92056 | **9748** | 94630 | 451928 |
| IL_KANE | 97118 | 48883 | **10007** | 716906 | 177742 | **28036** | 1529117 | **193514** | 214938 |
| IL_LAKE | 22348 | 20659 | **3121** | 194850 | 150124 | **19432** | 392956 | 268646 | **75696** |
| IL_MCHENRY | 1541 | 971 | **105** | 20823 | 11986 | **2858** | 43753 | 24046 | **2303** |
| IL_WILL | 54643 | **17536** | 28639 | 683068 | 252364 | **195247** | 1302020 | **467553** | 503499 |
| IL_WINNEBAGO | 12008 | 25663 | **637** | 104533 | 63657 | **3476** | 189777 | 89217 | **18648** |
| IN_ALLEN | 3113 | 2207 | **399** | 54806 | 39605 | **11844** | 141443 | 103276 | **27724** |
| IN_CASS | 4281 | 51192 | **161** | 61903 | 244562 | **39374** | 121784 | 373554 | **82848** |
| IN_LAKE | 5620 | 10588 | **94** | 46336 | 88827 | **814** | 97882 | 182628 | **1953** |
| IN_MARION | 114294 | 91430 | **6926** | 1132428 | 892047 | **173817** | 2387723 | 1855274 | **402497** |
| IN_ST. JOSEPH | 1644 | 2559 | **279** | **5614** | 9194 | 7379 | **6187** | 10926 | 9092 |
| KS_FINNEY | 1756 | **413** | 1383 | 6518 | **394** | 87271 | 5355 | **342** | 265359 |
| KS_FORD | 735 | **386** | 1812 | 22771 | 5636 | **947** | 60373 | 15549 | **2929** |
| KS_LEAVENWORTH | 48028 | 11465 | **2948** | 230442 | 32594 | **2929** | 337744 | 42241 | **2240** |
| KS_WYANDOTTE | 5054 | **64** | 222 | 77379 | 8115 | **220** | 156149 | 23969 | **594** |
| KY_JEFFERSON | 10906 | 10230 | **1502** | 85694 | 82463 | **6270** | 220004 | 213616 | **23916** |
| MA_BARNSTABLE | 387 | 752 | **213** | 9777 | 15033 | **934** | 29253 | 40907 | **1074** |
| MA_BRISTOL | 12514 | 51827 | **2235** | **6480** | 102874 | 97789 | **9714** | 181026 | 223140 |
| MA_ESSEX | 151851 | 90157 | **1234** | 1763177 | 1101095 | **4725** | 4300938 | 2854118 | **9959** |
| MA_HAMPDEN | 5641 | 7548 | **4033** | 62516 | 51060 | **48710** | 143037 | 98799 | **60158** |
| MA_MIDDLESEX | 199142 | 55108 | **1296** | 1971598 | 302943 | **19451** | 5544507 | 1129888 | **37912** |
| MA_NORFOLK | 14639 | 22658 | **2028** | 85585 | 85928 | **8694** | 370634 | 326201 | **17925** |
| MA_PLYMOUTH | 24836 | 41936 | **6342** | 156822 | 136391 | **3274** | 460709 | 325898 | **5710** |
| MA_SUFFOLK | 312397 | 332126 | **124583** | 1328344 | 1002098 | **399111** | 3053775 | 2057393 | **609131** |
| MA_WORCESTER | 230221 | 177247 | **4152** | 944588 | 563306 | **151225** | 1838511 | 1041356 | **391841** |
| MD_ANNE ARUNDEL | 2008 | 703 | **429** | 54287 | 25237 | **7824** | 153478 | 75802 | **14848** |
| MD_BALTIMORE | 91553 | 88639 | **18452** | 449595 | 407736 | **67106** | 985647 | 878925 | **126229** |
| MD_FREDERICK | 6510 | 1259 | **1113** | 86343 | 17892 | **12038** | 171214 | 31956 | **18727** |
| MD_HOWARD | 7625 | 7152 | **3344** | 107564 | 98018 | **20722** | 209720 | 188126 | **35380** |
| MD_MONTGOMERY | 179736 | 210302 | **124718** | 1391033 | 1562926 | **413167** | 2938922 | 3121218 | **535881** |
| MD_PRINCE GEORGE'S | 397082 | 579986 | **86136** | 3018645 | 4057905 | **177241** | 6012276 | 7491443 | **142804** |
| MI_GENESEE | 1680 | 1905 | **764** | 7053 | 6005 | **3729** | 13014 | 9712 | **6781** |
| MI_KENT | 10762 | 32952 | **621** | 113605 | 278827 | **25423** | 223533 | 506274 | **83847** |
| MI_MACOMB | **991** | 13931 | 6198 | **518** | 51568 | 4545 | **591** | 103669 | 6493 |
| MI_OAKLAND | 12869 | 22483 | **4005** | 28394 | 89500 | **23302** | 47459 | 166421 | **30410** |
| MI_WASHTENAW | **17** | 26 | 316 | **21** | 238 | 253 | **19** | 566 | 605 |
| MI_WAYNE | 83269 | 238758 | **7091** | **144660** | 783108 | 250736 | **202432** | 1294491 | 360187 |
| MN_HENNEPIN | 232924 | 471990 | **8277** | 2828055 | 1748628 | **1232435** | 5721830 | 2512438 | **974097** |
| MN_NOBLES | 4361 | 7410 | **50** | 71850 | 65120 | **15335** | 153882 | 123290 | **31593** |
| MN_RAMSEY | 72683 | 139518 | **472** | 750768 | 592492 | **243283** | 1441383 | 896080 | **223839** |
| MN_STEARNS | 99819 | 8391 | **522** | 714423 | **29040** | 61067 | 1100175 | **38931** | 202698 |
| MO_ST. LOUIS | 25750 | 23919 | **888** | 238588 | 229732 | **25322** | 501347 | 487804 | **38271** |
| NC_DURHAM | 1330 | 4449 | **594** | 41014 | 89773 | **26102** | 109789 | 220458 | **73622** |
| NC_MECKLENBURG | 20347 | 23153 | **18660** | **212885** | 233534 | 323133 | **590732** | 638547 | 864899 |
| NC_WAKE | 1584 | 6970 | **381** | 17082 | 70686 | **14369** | 44968 | 162871 | **38007** |
| ND_CASS | **5048** | 9190 | 5305 | **14637** | 34727 | 50176 | **11731** | 36265 | 51899 |
| NE_DAKOTA | 19069 | 2246 | **528** | 69683 | 77939 | **1012** | 117925 | 152069 | **798** |
| NE_DOUGLAS | 65806 | 122970 | **3088** | 725611 | **473570** | 1669426 | 1829956 | **939132** | 2662947 |
| NE_HALL | 9019 | 10992 | **4240** | 59916 | 64759 | **14255** | 117316 | 121579 | **23566** |
| NH_HILLSBOROUGH | 3958 | 967 | **903** | 54748 | **496** | 26902 | 143957 | **2444** | 26357 |
| NH_ROCKINGHAM | 9667 | 8885 | **3931** | 62103 | 58838 | **1455** | 108403 | 103667 | **1163** |
| NJ_ATLANTIC | 5606 | 7598 | **1060** | 74334 | 68545 | **2620** | 174340 | 144191 | **2462** |
| NJ_BERGEN | 57602 | 43609 | **14097** | 749463 | 432140 | **38632** | 1562395 | 833936 | **47132** |
| NJ_BURLINGTON | 20886 | 7370 | **5470** | 359663 | 199200 | **12902** | 742457 | 443681 | **39671** |
| NJ_CAMDEN | 74153 | 32353 | **16478** | 770110 | 359467 | **4550** | 1594326 | 776531 | **18626** |
| NJ_CUMBERLAND | 28008 | **6203** | 19126 | 260015 | **39695** | 53378 | 536407 | 86277 | **72420** |
| NJ_ESSEX | 314563 | 250949 | **23589** | 1895021 | 1464297 | **41255** | 3590038 | 2806264 | **31434** |
| NJ_GLOUCESTER | 10695 | 10789 | **814** | 95772 | 87840 | **1827** | 183775 | 163840 | **10935** |
| NJ_HUDSON | 570528 | 148403 | **49027** | 2686119 | **235003** | 3990343 | 5034222 | **344182** | 7551543 |
| NJ_MERCER | 121380 | 67124 | **12611** | 1168866 | 753970 | **33796** | 2278538 | 1553999 | **41029** |
| NJ_MIDDLESEX | 184452 | 34017 | **21067** | 2220576 | 586433 | **1778** | 4615178 | 1322828 | **8728** |
| NJ_MONMOUTH | 1204 | 14276 | **420** | 38122 | 109691 | **20080** | 90151 | 202365 | **25572** |
| NJ_MORRIS | 5817 | 7434 | **214** | 87317 | 61142 | **7477** | 229380 | 145189 | **8670** |
| NJ_OCEAN | 38914 | 10048 | **657** | 449643 | 91232 | **500** | 943645 | 183766 | **759** |
| NJ_PASSAIC | 198220 | 98352 | **48633** | 1878404 | 934699 | **116003** | 3866673 | 1965730 | **146634** |
| NJ_SOMERSET | 9485 | **2282** | 3035 | 118735 | 33769 | **475** | 242719 | 70106 | **1943** |
| NJ_UNION | 230920 | 142931 | **11723** | 1201539 | 478223 | **29120** | 2486633 | 932038 | **35648** |
| NM_BERNALILLO | 3775 | 2404 | **441** | 39977 | 24274 | **342** | 84491 | 51512 | **275** |
| NM_MCKINLEY | 12433 | 12249 | **221** | 78035 | 68149 | **45594** | 163865 | 137964 | **57562** |
| NM_SAN JUAN | 2068 | **52** | 375 | 34109 | **547** | 32519 | 94078 | **1259** | 45719 |
| NV_CLARK | **15571** | 27908 | 42104 | 100213 | 179330 | **30981** | 177595 | 317077 | **34806** |
| NV_WASHOE | 9830 | 9690 | **3495** | 31980 | 32303 | **7311** | 57602 | 59071 | **11465** |
| NY_ALBANY | 2118 | 13031 | **981** | 11220 | 84838 | **3900** | 16341 | 141664 | **3328** |
| NY_DUTCHESS | 12570 | 16050 | **5295** | 51028 | 69479 | **21483** | 81019 | 111836 | **21629** |
| NY_ERIE | 28411 | 26691 | **1294** | 561542 | 529422 | **28862** | 1103128 | 1032425 | **27138** |
| NY_MONROE | 29634 | 36475 | **11366** | **66258** | 86075 | 71718 | **70156** | 94745 | 82125 |
| NY_NASSAU | 40901 | 222796 | **5613** | 553854 | 1525527 | **259827** | 1378727 | 3054513 | **415975** |
| NY_NEW YORK | **4481900** | 13736222 | 5796699 | 38997234 | 119978471 | **732772** | 86293738 | 252562616 | **841299** |
| NY_ONONDAGA | 7404 | **5925** | 8277 | 90287 | **42704** | 74655 | 165223 | **60091** | 93781 |
| NY_ORANGE | 10580 | 68098 | **612** | 114560 | 561972 | **65925** | 247114 | 1101615 | **92361** |
| NY_PUTNAM | **374** | 2943 | 775 | 11849 | 36610 | **6936** | 24988 | 66187 | **8877** |
| NY_ROCKLAND | 24740 | 75876 | **2819** | 219648 | 425775 | **63111** | 471005 | 779100 | **91923** |
| NY_SUFFOLK | 34498 | 140823 | **5325** | 264714 | 704596 | **92010** | 757294 | 1477103 | **136150** |
| NY_SULLIVAN | 1647 | 3104 | **82** | 7188 | 10222 | **299** | 14698 | 17193 | **1090** |
| NY_ULSTER | 1498 | 6383 | **136** | 8002 | 38172 | **3758** | 15220 | 69847 | **5105** |
| NY_WESTCHESTER | 96710 | 222021 | **3275** | 995981 | 1321971 | **58596** | 2158805 | 2442246 | **73782** |
| OH_CUYAHOGA | 6715 | 10468 | **2089** | 58702 | 111572 | **34729** | 104111 | 215825 | **48039** |
| OH_FRANKLIN | 24100 | 2330 | **1937** | 555182 | 113725 | **3181** | 1319032 | 285580 | **16555** |
| OH_HAMILTON | 633 | 1475 | **168** | 37592 | 54589 | **19322** | 77150 | 108339 | **21984** |
| OH_LUCAS | **16** | 2611 | 41 | 12000 | **4091** | 9978 | 40863 | **3088** | 20046 |
| OH_MAHONING | 3402 | 686 | **230** | 51850 | 22879 | **95** | 100764 | 49053 | **114** |
| OH_MARION | 3999 | 6761 | **1985** | 17235 | 40475 | **1575** | 24586 | 67858 | **1312** |
| OH_PICKAWAY | **567** | 1498 | 1128 | **8296** | 18504 | 15377 | **11791** | 28936 | 22152 |
| OK_OKLAHOMA | 583 | **274** | 367 | 2141 | 1010 | **305** | 2398 | 1012 | **568** |
| PA_ALLEGHENY | 503 | 1316 | **240** | 5469 | 10714 | **590** | 9353 | 17768 | **583** |
| PA_BERKS | 2883 | 1272 | **569** | 7411 | **6162** | 81962 | **5811** | 6285 | 124454 |
| PA_BUCKS | 3222 | **222** | 3121 | 29363 | **1394** | 62365 | 65786 | **2465** | 156482 |
| PA_CHESTER | 365 | **290** | 322 | **360** | 10184 | 472 | **1899** | 37323 | 3464 |
| PA_DELAWARE | 14767 | 5882 | **2096** | 50456 | **5282** | 231007 | 80729 | **5583** | 530827 |
| PA_LACKAWANNA | 4221 | 4284 | **4064** | 13369 | 13112 | **1705** | 18486 | 17710 | **1969** |
| PA_LANCASTER | 4214 | 4833 | **1204** | 70586 | 73934 | **928** | 125565 | 129860 | **1027** |
| PA_LEHIGH | 577 | **434** | 557 | 1394 | **507** | 510 | 2009 | **943** | 1433 |
| PA_LUZERNE | 106 | **57** | 1187 | 2867 | 1046 | **426** | 6460 | 1848 | **1640** |
| PA_MONROE | 30 | 203 | **28** | **590** | 3477 | 1014 | 1392 | 7438 | **1243** |
| PA_MONTGOMERY | 19107 | 36128 | **13765** | 48071 | 127458 | **23246** | 63607 | 193276 | **43896** |
| PA_NORTHAMPTON | 19449 | 21644 | **3604** | 96572 | 105765 | **3751** | 166719 | 180823 | **3296** |
| PA_PHILADELPHIA | 8900 | 73311 | **5543** | 133723 | 2083975 | **118964** | 389486 | 4888068 | **246007** |
| RI_PROVIDENCE | 73961 | **72896** | 79131 | **48303** | 75328 | 1156807 | 156014 | **73946** | 1406586 |
| SC_GREENVILLE | **953** | 1329 | 2150 | **5768** | 7762 | 7824 | **32151** | 37418 | 35045 |
| SC_RICHLAND | **87** | 1589 | 115 | 898 | 7809 | **529** | 2657 | 15175 | **1129** |
| SD_MINNEHAHA | 54066 | 45168 | **1670** | 329942 | 270958 | **51358** | 552250 | 443211 | **86124** |
| TN_DAVIDSON | 38256 | 22294 | **8664** | 409129 | 223940 | **114675** | 859989 | 471462 | **159944** |
| TN_SHELBY | 17083 | 19805 | **5827** | 282417 | 301841 | **157875** | 680238 | 709492 | **328022** |
| TN_TROUSDALE | 49779 | 5354 | **156** | 256031 | 7716 | **18** | 390612 | 22600 | **36** |
| TX_BEXAR | 71067 | 10834 | **9870** | 406311 | **29116** | 75931 | 810178 | **74296** | 129339 |
| TX_COLLIN | 221 | 88 | **33** | 6610 | **355** | 894 | 20069 | **1939** | 2613 |
| TX_DALLAS | 38824 | 86433 | **9751** | 801790 | 252512 | **183019** | 2605412 | 629107 | **306634** |
| TX_DENTON | 859 | 1303 | **94** | 4339 | 6076 | **1971** | 7774 | 10463 | **4085** |
| TX_EL PASO | 13463 | 22830 | **384** | 80869 | 132910 | **3756** | 114309 | 196954 | **3839** |
| TX_FORT BEND | 11556 | 4067 | **901** | 82542 | 30466 | **2177** | 171286 | 71474 | **1082** |
| TX_HARRIS | 91412 | 42218 | **7786** | 382379 | 284220 | **212653** | 772127 | 715891 | **521219** |
| TX_POTTER | 473038 | 367722 | **308955** | 683673 | 286331 | **237608** | 919281 | 280822 | **183812** |
| TX_TARRANT | 27924 | **3022** | 3077 | 495088 | 78464 | **39756** | 852668 | 119766 | **89502** |
| TX_TRAVIS | 910 | **782** | 797 | 44523 | 41865 | **13562** | 132624 | 126234 | **36121** |
| UT_SALT LAKE | 4448 | 2279 | **364** | 73190 | 33692 | **1322** | 245358 | 124108 | **5439** |
| UT_UTAH | 4073 | 2978 | **34** | 76383 | 63891 | **737** | 190014 | 165528 | **4484** |
| VA_ARLINGTON | 4346 | 3918 | **449** | 53808 | 41769 | **5230** | 102038 | 73135 | **5269** |
| VA_CHESTERFIELD | **283** | 955 | 1041 | **1133** | 5933 | 10927 | **8221** | 26980 | 30916 |
| VA_FAIRFAX | 9448 | **4018** | 31774 | **35393** | 119066 | 302720 | **62463** | 251097 | 363120 |
| VA_HENRICO | 1474 | 4450 | **121** | 17522 | 37374 | **16079** | 48833 | 90051 | **43311** |
| VA_LOUDOUN | 2516 | 10393 | **1920** | 167229 | 275123 | **165805** | 319188 | 505620 | **257788** |
| VA_PRINCE WILLIAM | **407** | 2245 | 40121 | **21976** | 50889 | 240130 | **59936** | 109037 | 332086 |
| WA_KING | 7945 | **1880** | 2126 | 43396 | 2345 | **1767** | 138388 | 17732 | **4438** |
| WA_PIERCE | 666 | 761 | **162** | 13399 | 16374 | **1039** | 34482 | 41971 | **3176** |
| WA_SNOHOMISH | 952 | 1483 | **6** | 14203 | 22925 | **518** | 31739 | 51899 | **852** |
| WA_YAKIMA | **17141** | 39890 | 49137 | **128400** | 190995 | 262414 | **369321** | 446671 | 533134 |
| WI_BROWN | 22808 | 5487 | **188** | 273075 | 109151 | **10094** | 500092 | 219092 | **29410** |
| WI_MILWAUKEE | 82335 | 81164 | **27184** | 776944 | 832194 | **537622** | 1373109 | 1511968 | **985075** |
| WI_RACINE | **7067** | 10089 | 10165 | 94742 | 72718 | **1729** | 173955 | 110921 | **10764** |

In order to check the performance difference statistically, we conduct T-test between each two models. The p-values are shown in below tables:

**Table 6.** P-values for state-level prediction (5-days)

| Model | SIR | SEIR | GRU | ColaGNN | CovidGNN | STAN |
| --- | --- | --- | --- | --- | --- | --- |
| SIR | 1.00E+00 | 5.93E-01 | 7.24E-05 | 1.40E-06 | 5.19E-06 | 0.00E+00 |
| SEIR | 5.93E-01 | 1.00E+00 | 1.31E-30 | 9.90E-73 | 1.16E-32 | 0.00E+00 |
| GRU | 7.24E-05 | 1.31E-30 | 1.00E+00 | 8.05E-08 | 1.66E-03 | 5.27E-16 |
| ColaGNN | 1.40E-06 | 9.90E-73 | 8.05E-08 | 1.00E+00 | 8.86E-02 | 9.54E-09 |
| CovidGNN | 5.19E-06 | 1.16E-32 | 1.66E-03 | 8.86E-02 | 1.00E+00 | 2.02E-12 |
| STAN | 5.71E-08 | 0.00E+00 | 5.27E-16 | 2.02E-12 | 9.54E-09 | 1.00E+00 |

**Table 7.** P-values for state-level prediction (15-days)

| Model | SIR | SEIR | GRU | ColaGNN | CovidGNN | STAN |
| --- | --- | --- | --- | --- | --- | --- |
| SIR | 1.00E+00 | 4.40E-142 | 1.62E-18 | 2.15E-54 | 3.76E-41 | 0.00E+00 |
| SEIR | 4.40E-142 | 1.00E+00 | 2.23E-03 | 3.96E-19 | 1.27E-07 | 0.00E+00 |
| GRU | 1.62E-18 | 2.23E-03 | 1.00E+00 | 2.06E-02 | 8.51E-01 | 3.46E-12 |
| ColaGNN | 2.15E-54 | 3.96E-19 | 2.06E-02 | 1.00E+00 | 3.23E-05 | 4.08E-20 |
| CovidGNN | 3.76E-41 | 1.27E-07 | 8.51E-01 | 3.23E-05 | 1.00E+00 | 4.38E-24 |
| STAN | 0.00E+00 | 0.00E+00 | 3.46E-12 | 4.08E-20 | 4.38E-24 | 1.00E+00 |

**Table 8.** P-values for state-level prediction (20-days)

| Model | SIR | SEIR | GRU | ColaGNN | CovidGNN | STAN |
| --- | --- | --- | --- | --- | --- | --- |
| SIR | 1.00E+00 | 2.15E-131 | 1.42E-28 | 1.87E-31 | 7.68E-27 | 0.00E+00 |
| SEIR | 2.15E-131 | 1.00E+00 | 5.56E-10 | 6.65E-14 | 4.40E-09 | 4.04E-263 |
| GRU | 1.42E-28 | 5.56E-10 | 1.00E+00 | 7.69E-02 | 9.04E-01 | 2.70E-08 |
| ColaGNN | 1.87E-31 | 6.65E-14 | 7.69E-02 | 1.00E+00 | 6.61E-02 | 1.03E-04 |
| CovidGNN | 7.68E-27 | 4.40E-09 | 9.04E-01 | 6.61E-02 | 1.00E+00 | 5.27E-08 |
| STAN | 0.00E+00 | 4.04E-263 | 2.70E-08 | 1.03E-04 | 5.27E-08 | 1.00E+00 |

**Table 9.** P-values for county-level prediction (5-days)

| Model | SIR | SEIR | GRU | ColaGNN | CovidGNN | STAN |
| --- | --- | --- | --- | --- | --- | --- |
| SIR | 1.00E+00 | 2.30E-13 | 5.52E-03 | 2.67E-10 | 4.43E-04 | 0.00E+00 |
| SEIR | 2.30E-13 | 1.00E+00 | 2.30E-36 | 3.33E-81 | 1.67E-50 | 1.46E-203 |
| GRU | 5.52E-03 | 2.30E-36 | 1.00E+00 | 9.00E-06 | 4.55E-01 | 8.46E-18 |
| ColaGNN | 2.67E-10 | 3.33E-81 | 9.00E-06 | 1.00E+00 | 3.36E-05 | 2.83E-07 |
| CovidGNN | 4.43E-04 | 1.67E-50 | 4.55E-01 | 3.36E-05 | 1.00E+00 | 3.07E-20 |
| STAN | 8.91E-20 | 1.46E-203 | 8.46E-18 | 2.83E-07 | 3.07E-20 | 1.00E+00 |

**Table 10.** P-values for county-level prediction (15-days)

| Model | SIR | SEIR | GRU | ColaGNN | CovidGNN | STAN |
| --- | --- | --- | --- | --- | --- | --- |
| SIR | 1.00E+00 | 2.52E-18 | 1.24E-49 | 2.21E-88 | 4.75E-42 | 0.00E+00 |
| SEIR | 2.52E-18 | 1.00E+00 | 1.25E-78 | 4.86E-116 | 2.29E-71 | 6.21E-253 |
| GRU | 1.24E-49 | 1.25E-78 | 1.00E+00 | 2.22E-12 | 6.08E-01 | 3.57E-84 |
| ColaGNN | 2.21E-88 | 4.86E-116 | 2.22E-12 | 1.00E+00 | 4.00E-09 | 5.23E-60 |
| CovidGNN | 4.75E-42 | 2.29E-71 | 6.08E-01 | 4.00E-09 | 1.00E+00 | 4.32E-69 |
| STAN | 0.00E+00 | 6.21E-253 | 3.57E-84 | 5.23E-60 | 4.32E-69 | 1.00E+00 |

**Table 11.** P-values for county-level prediction (20-days)

| Model | SIR | SEIR | GRU | ColaGNN | CovidGNN | STAN |
| --- | --- | --- | --- | --- | --- | --- |
| SIR | 1.00E+00 | 7.48E-18 | 2.15E-96 | 1.25E-163 | 2.14E-83 | 0.00E+00 |
| SEIR | 7.48E-18 | 1.00E+00 | 3.41E-123 | 1.69E-166 | 5.02E-111 | 9.74E-257 |
| GRU | 2.15E-96 | 3.41E-123 | 1.00E+00 | 6.44E-06 | 6.19E-02 | 5.28E-56 |
| ColaGNN | 1.25E-163 | 1.69E-166 | 6.44E-06 | 1.00E+00 | 1.40E-10 | 9.85E-57 |
| CovidGNN | 2.14E-83 | 5.02E-111 | 6.19E-02 | 1.40E-10 | 1.00E+00 | 2.57E-61 |
| STAN | 0.00E+00 | 9.74E-257 | 5.28E-56 | 9.85E-57 | 2.57E-61 | 1.00E+00 |

The results indicate the performance of STAN have significant difference with baseline models (p-value << 0.05), and STAN performs statistically better than all baselines.
